# Automated bone age assessment in rare pediatric growth disorders: a comparative study using Deeplasia

**DOI:** 10.3389/fendo.2026.1741927

**Published:** 2026-02-06

**Authors:** Kyra Skaf, Minu Fardipour, Philipp Schmidt, Eike Bolmer, Alexandra Keller, Christina Lampe, Julian Jurgens, Mona Lindschau, Katja Palm, Sophie Ruckdeschel, Behnam Javanmardi, Klaus Mohnike

**Affiliations:** 1 Medical Faculty, Otto-Von-Guericke-University Magdeburg, Magdeburg, Germany; 2 Institute for Genomic Statistics and Bioinformatics, University Hospital Bonn, Bonn, Germany; 3 Kinderzentrum Am Johannisplatz, Leipzig, Germany; 4 Centre for Rare Diseases, University Hospital of Giessen, Giessen, Germany; 5 Division of Pediatric Radiology, Department of Radiology, University Hospital Hamburg, Hamburg, Germany; 6 International Center for Lysosomal Disorders (ICLD), Department of Pediatrics, University Medical Center Hamburg-Eppendorf, Hamburg, Germany

**Keywords:** bone age, rare growth disorders, artificial intelligence, pediadtric radiology, deeplasia, Greulich and Pyle method

## Abstract

**Objective:**

Bone age (BA) assessment is essential for monitoring growth and maturation and guiding therapeutic interventions. While deep learning (DL) models offer high-speed automated BA prediction, their generalizability to rare pathological and diagnostically complex populations remains a significant concern. This study aims to validate the open-source DL system Deeplasia on external data from pediatric patients with various syndromic, endocrine, and lysosomal storage disorders (LSDs) and to compare its accuracy and consistency against multiple expert human raters.

**Methods:**

We retrospectively assembled 1,138 hand radiographs from multiple centers, including patients with SHOX deficiency; Noonan syndrome; Silver–Russell syndrome; Ullrich–Turner syndrome; pseudohypoparathyroidism; congenital adrenal hyperplasia (CAH); precocious puberty and precocious pseudopuberty (cohort 1); mucopolysaccharidosis types I, II, III, IV, and VI; alpha-mannosidosis; and unclassified LSDs (cohort 2). For each radiograph, BA was evaluated using the Greulich and Pyle method by two to five human experts to obtain a mean BA reference. Model performance was assessed using the mean absolute error (MAE), root mean squared error (RMSE), and 1-year accuracy for each cohort and underlying conditions, sex, and age groups. Furthermore, Deeplasia’s performance was compared with that of individual raters by testing each rater and the model against the remaining experts.

**Results:**

Deeplasia achieved a mean MAE of 5.95 months, an RMSE of 8.01 months, and a 1-year accuracy of 89.9% for cohort 1 (endocrine and syndromic conditions). For cohort 2 (lysosomal storage disorders), Deeplasia achieved a mean MAE of 7.13 months, an RMSE of 9.56 months, and a 1-year accuracy of 81.2%. In direct comparisons between Deeplasia and individual raters tested against the remaining experts, Deeplasia outperformed all human raters.

**Conclusion:**

Deeplasia was validated as a highly consistent, robust, and reliable tool for BA assessment in complex cases. It demonstrated superior accuracy compared with individual human raters and may assist clinicians in BA evaluation.

## 1 Introduction

Bone age (BA) assessment is a fundamental tool in pediatrics, orthopedics, and forensic medicine, providing a proxy for skeletal maturity crucial for diagnosing growth disorders, monitoring pubertal development, and guiding therapeutic decisions such as growth hormone treatment (1–14). Delayed ossification may indicate underlying nutritional deficiency, endocrine dysfunction, or developmental disorders, whereas accelerated maturation may signal precocious puberty or other endocrine pathologies. As such, this method may serve as a practical screening tool for pediatricians to identify children requiring further evaluation or referral. Reliable and reproducible methods for BA assessment have been pursued for decades (15–18). Traditionally, BA is evaluated using left-hand and wrist radiographs via atlas-based methods such as the Greulich–Pyle (GP) atlas (19) or scoring-based methods like the Tanner–Whitehouse (TW) system (17, 18, 20). While the GP method is widely used due to its relative simplicity, it is inherently subjective and prone to significant inter- and intraobserver variability (9). TW methods are also time-intensive, limiting their efficiency in busy clinical workflows. The rapid evolution of deep learning (DL) and convolutional neural networks (CNNs) has since transformed the field, especially following the 2017 RSNA Pediatric Bone Age Challenge (21). Modern CNN-based approaches consistently achieve mean absolute errors (MAEs) often below 6 months, in many cases outperforming traditional manual scoring methods (3, 4, 14, 22, 23). Crucially, the fully automated nature of these systems offers a substantial gain in clinical workflow efficiency, reducing assessment time from minutes to seconds. The intense drive for automated, objective assessment has fostered the development of a wide range of specialized deep learning architectures (24–28). Beyond simply automating existing methods, research has explored advanced CNN applications to maximize accuracy and robustness: Architectures such as MABAL (machine-assisted bone age labeling) (28) and models utilizing regression CNNs (24) have been employed for direct age estimation. Other advanced methods have focused on improving performance by mimicking the radiologist’s diagnostic workflow using stacked neural networks (26) or by optimizing feature extraction from the entire radiograph (27). This collective effort, including performance evaluations across large datasets (28), highlights the field’s vigorous pursuit of reliable and scalable AI solutions. Despite these advances, most AI models for BA assessment are predominantly trained and validated on standardized datasets such as the RSNA challenge set, which primarily includes images from healthy populations (21–23, 29). Consequently, these models may exhibit reduced generalizability when applied to real-world clinical radiographs, particularly those from pediatric patients with underlying pathological skeletal changes (30), growth disorders (22, 23, 31), or dysplasias (32, 33). To address this critical limitation, Rassmann et al. (34) introduced Deeplasia, an open-source deep learning-based BA assessment system specifically validated on skeletal dysplasias. In the present work, we evaluate the performance of Deeplasia on two external, diagnostically complex clinical cohorts with various syndromic, endocrine, and lysosomal storage disorders. By comparing Deeplasia against multiple expert human raters, this paper aims to rigorously assess the system’s robustness, accuracy, and reliability under real-world clinical conditions and to demonstrate its potential for effective and efficient deployment in specialized pediatric radiology.

## 2 Materials and methods

### 2.1 Study design and cohorts

The study included two pediatric cohorts: cohort 1, comprising syndromic and endocrine disorders, and cohort 2, comprising lysosomal storage disorders. Radiographs were evaluated by human raters using the Greulich–Pyle method. In cohort 1, each radiograph was independently assessed by up to three raters, while in cohort 2, evaluations were performed by five raters. The panel of raters intentionally covered a broad spectrum of clinical expertise, ranging from medical students and residents in pediatrics to senior pediatric endocrinologists with more than 30 years of clinical experience. This design allowed for the assessment of interrater variability across different levels of training. In parallel, BA was also predicted by Deeplasia, a deep learning-based algorithm, which provided an independent reference estimate of skeletal maturity. For comparative analyses, patients were stratified by sex and further subdivided into four age groups based on chronological age at the time of imaging: 1) younger than 48 months (<4 years), 2) 48–96 months (4–8 years), 3) 96–144 months (8–12 years), and 4) older than 144 months (>12 years). This stratification was chosen to reflect clinically relevant developmental stages of childhood and adolescence, thereby facilitating both sex-specific and age-specific comparisons of bone maturation patterns across diagnostic groups. Because patient age at the time of image acquisition was not available for all images, complete age group assignment was not possible; therefore, all patients with unknown chronological age were assigned to an additional group 5.

#### 2.1.1 Cohort 1

This cohort contains 950 left-hand radiographs from patients with congenital adrenal hyperplasia (*n* = 95), precocious pseudopuberty (*n* = 11), precocious puberty (*n* = 96), pseudohypoparathyroidism (*n* = 30), Noonan syndrome (*n* = 116), SHOX deficiency (*n* = 292), Silver–Russell syndrome (*n* = 69), and Ullrich–Turner syndrome (*n* = 241) diagnosed and treated at the University Hospital of Magdeburg and a specialized pediatric endocrinology consultation at Leipzig, Germany. Radiographs were acquired between 2006 and 2024. Patient ages at the time of imaging spanned from 1.1 to 18.5 years. The sex distribution was 580 female patients and 370 male patients. The distribution in age groups was *n* = 51 for group 1, *n* = 216 for group 2, *n* = 261 for group 3, and *n* = 220 for group 4.

#### 2.1.2 Cohort 2

This cohort comprises 186 hand radiographs from patients with mucopolysaccharidosis (MPS) I (*n* = 31), MPS II (*n* = 19), MPS III (*n* = 24), MPS IV (*n* = 28), MPS VI (*n* = 15), alpha-mannosidosis (*n* = 26), and unclassified lysosomal storage diseases (*n* = 43). Pseudonymized radiographs were collected from the pediatric departments in Hamburg-Eppendorf (2009–2022), Münster (2010–2015), Giessen (2016–2022), and Bochum (from 2014). In addition, a large collection was provided by Prof. J. Spranger, whose archive contains historic patient records from 1961 to 1988. Patient ages at the time of imaging spanned from 0 to 40 years. The sex distribution was 69 female patients and 117 male patients. The distribution in age groups was *n* = 46 for group 1, *n* = 48 for group 2, *n* = 26 for group 3, and *n* = 30 for group 4.

### 2.2 AI system: Deeplasia

Deeplasia (34) is a state-of-the-art prior-free deep convolutional neural network developed for the fully automated estimation of skeletal maturity from pediatric hand radiographs (34). In contrast to traditional atlas-based or explicit region-of-interest (ROI) approaches, the system utilizes an end-to-end learning strategy that enables it to capture both global and local image features in a data-driven manner, without relying on handcrafted priors. This design choice is critical for generalizing to atypical skeletal morphology, frequently encountered in patients with syndromic and dysplastic disorders. The core models were trained on the Radiological Society of North America (RSNA) Pediatric Bone Age dataset, which comprises over 14,000 radiographs, utilizing the Greulich–Pyle method as the consensus reference standard. The network architecture is based on EfficientNet variants. To account for known sex-specific maturation patterns, Deeplasia integrates sex information as a binary covariate, embedded and concatenated with the image features prior to the final regression. The system operates as a robust ensemble of three deep CNN variants derived from the described architecture. The final prediction is generated as the mean of these three independent model estimates. The process includes an initial automated hand-segmentation step to normalize image input and minimize the influence of artifacts, labels, or borders. As an open-source AI, the entire pipeline, including preprocessing masks and source code, has been made publicly available (34) to ensure transparency and reproducibility. Deeplasia has been previously validated on external clinical cohorts, demonstrating high test–retest precision suitable for longitudinal applications and high accuracy in patients with genetically confirmed skeletal dysplasias (32, 34).

### 2.3 Statistical analysis

**Figure 1:**
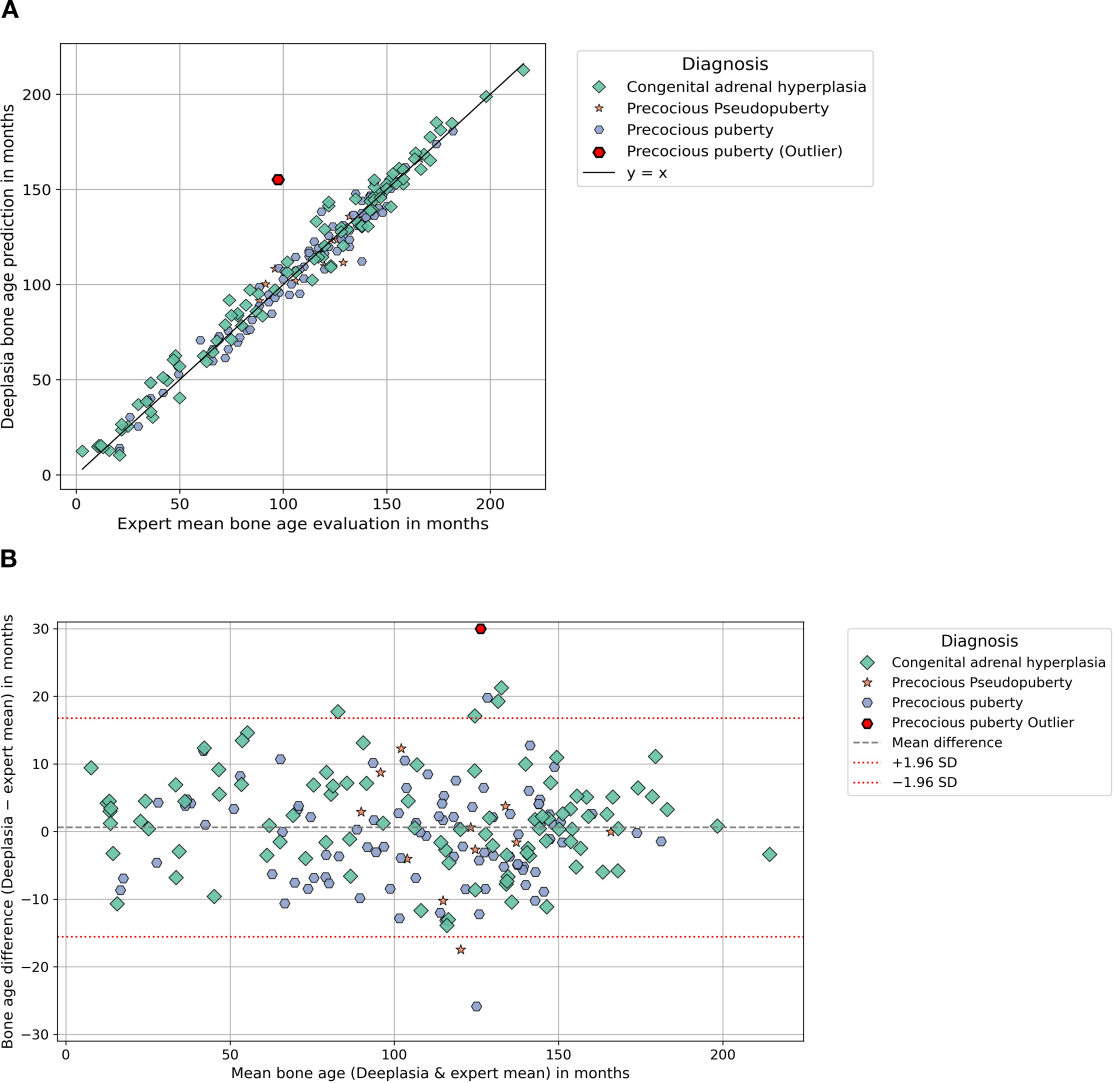
Comparing the performance of Deeplasia and expert raters in cohort 1 “syndromic and endocrine disorders” for conditions with accelerated bone age. Panel **(A)** shows a scatter plot comparing Deeplasia bone age predictions with expert evaluations for disorders with accelerated growth, with some outliers. Panel **(B)** shows the corresponding Bland-Altman plot, displaying the difference between Deeplasia and the expert evaluations plotted against their average. Horizontal lines indicate the mean bias and limits of agreement, with a small number of outliers.

**Figure 2:**
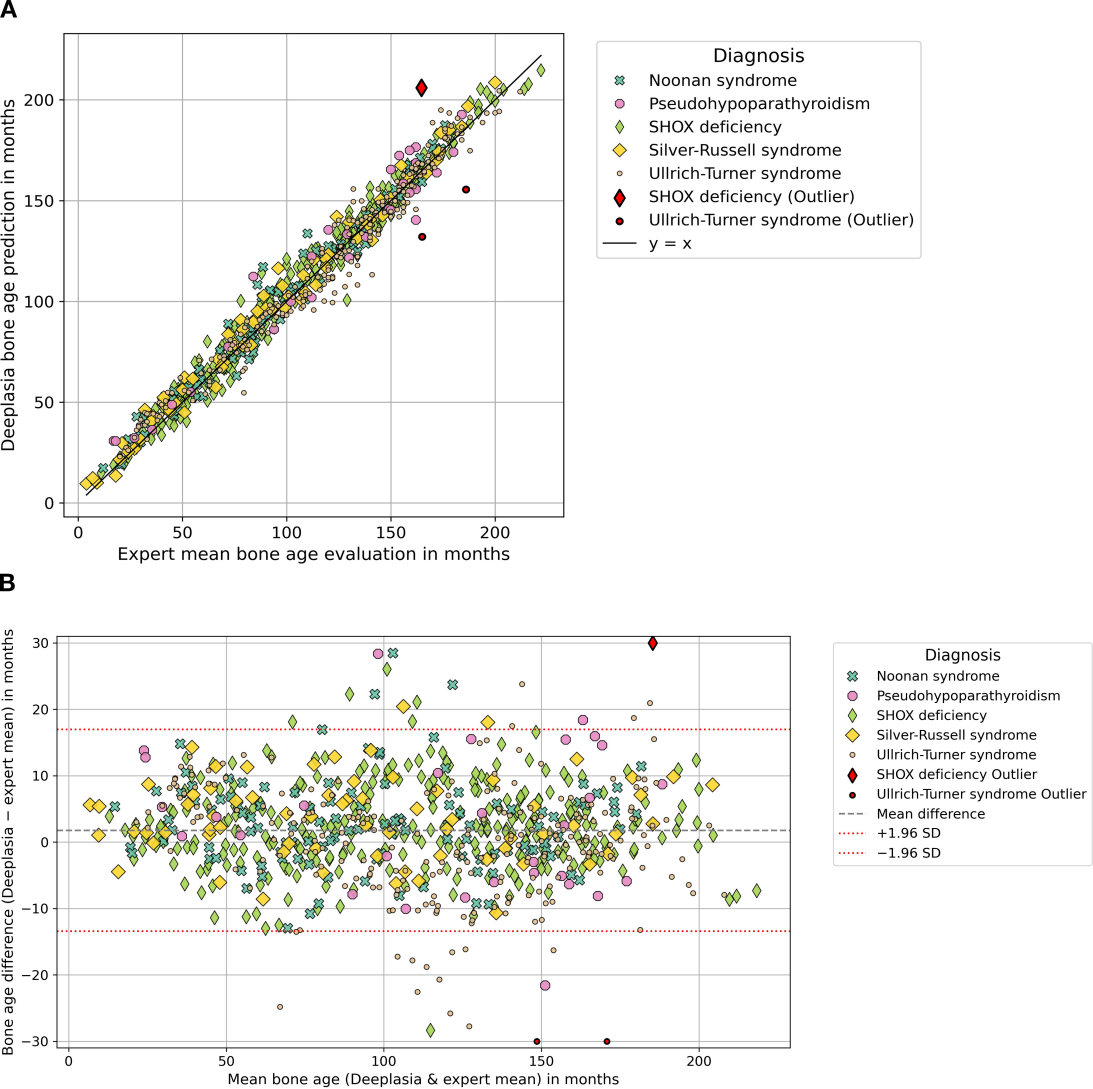
Comparing the performance of Deeplasia and expert raters in cohort 1 “syndromic and endocrine disorders” for conditions with decelerated bone age. Panel **(A)** shows a scatter plot comparing Deeplasia bone age predictions with expert evaluations for conditions with delayed growth, with several outliers. Panel **(B)** shows the corresponding Bland-Altman plot, displaying the difference between Deeplasia and the expert evaluations plotted against their average. Horizontal lines indicate the mean bias and limits of agreement, with a small number of outliers.

**Figure 3:**
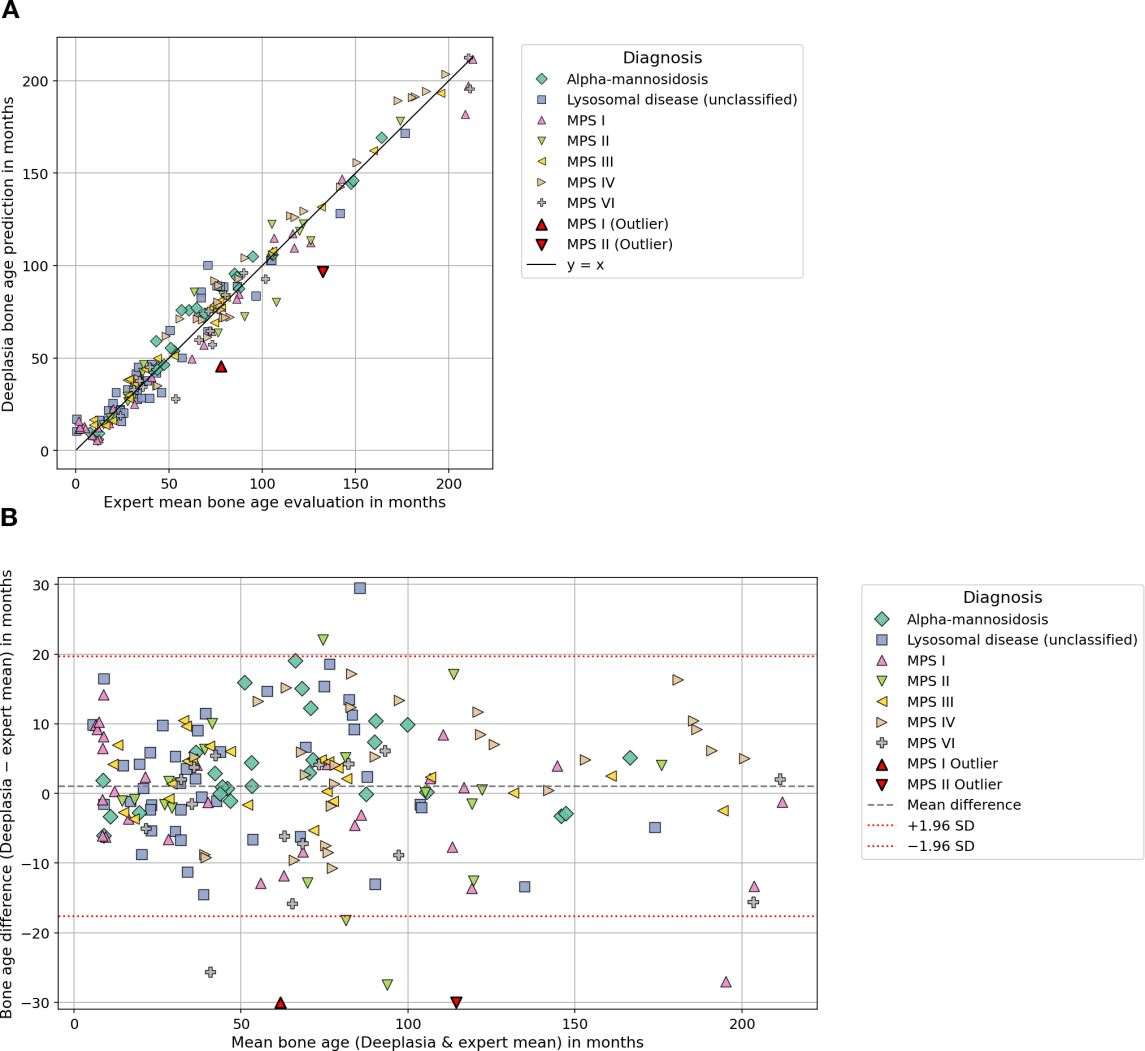
Comparing the performance of Deeplasia and expert raters in cohort 2 “lysosomal storage disorders”. Panel **(A)** shows a scatter plot comparing Deeplasia bone age predictions with expert evaluations in patients with lysosomal storage disorders, including different mucopolysaccharidosis subtypes and related conditions. A small number of outliers is visible. Panel **(B)** shows the corresponding Bland-Altman plot, displaying the difference between Deeplasia and the expert evaluations plotted against their mean. Horizontal lines indicate the mean bias and limits of agreement, with a small number of outliers.

Accuracy was assessed using the MAE, root mean squared error (RMSE), and the proportion of predictions within ±12 months (1-year accuracy). The relationship between Deeplasia and the reference standard was graphically assessed using scatter plots and Bland–Altman analysis (see **Figures 1**-**3**). Subgroup analyses were conducted based on the diagnosis, sex, and age group definitions, outlined in Section 2.1 (see **Tables 1**, **2**). For cohort 1, while a complete review by three human raters was available for the subset of *n* = 849 images, the remaining 101 images were assessed by two raters, and all 950 images were included in the overall statistical analysis by using the mean BA of the available raters as the reference. To enable a fair comparison of the performance of Deeplasia with that of individual human raters, we employed a “leave-one-out” cross-validation procedure. Specifically, the mean BA assessed by the other experts served as the reference standard. We then computed the MAE, RMSE, and 1-year accuracy for both the excluded rater and Deeplasia, relative to this specific mean BA. This procedure was repeated for every individual rater in both cohorts (see **Tables 3**, **4**). All images and their corresponding BA estimations were included in the statistical analysis. Outliers, as indicated in **Figures 1**–**3**, were defined as images for which the absolute difference between the Deeplasia prediction and the mean expert consensus BA exceeded 30 months. These BA estimations and predictions were subsequently examined individually (see **Table 5**).

**Table 1:** Performance of Deeplasia across different subgroups in cohort 1 (syndromic and endocrine disorders).

Specific subgroup	*N* (images)	MAE	RMSE	1-Year accuracy (%)	Pearson correlation	Bias	Mean raters STD
Congenital adrenal hyperplasia	95	5.76	7.37	89.5	0.99	1.7	7.92
Noonan syndrome	116	5.47	7.34	91.4	0.98	2.8	8.28
Precocious pseudopuberty	11	5.85	7.9	81.8	0.93	−0.73	6.38
Precocious puberty	96	6.03	9.07	91.7	0.97	−0.32	9.62
Pseudohypoparathyroidism	30	9.10	11.11	70.0	0.98	3.15	11.88
SHOX deficiency	292	5.44	7.35	92.47	0.99	2.49	7.57
Silver–Russell syndrome	69	5.76	7.21	91.3	0.99	3.72	6.95
Ullrich–Turner syndrome	241	6.5	8.61	88.0	0.98	−0.35	8.47
Mean of all eight conditions	–	6.24	8.25	87.0	0.98	1.56	8.38
All images in cohort 1	950	5.95	8.01	89.9	0.98	1.52	7.87
Sex: F	580	6.04	8.18	90.0	0.98	0.31	8.18
Sex: M	370	5.8	7.73	89.7	0.99	3.41	6.95
Age: <4 years	51	3.76	5.1	96.1	0.89	3.23	3.99
Age: 4–8 years	216	5.88	7.26	91.7	0.93	2.1	6.97
Age: 8–12 years	261	6.78	9.02	85.8	0.91	0.82	9
Age: >12 years	220	5.59	7.77	91.4	0.93	2.22	7.46
Age: unknown	202	5.89	8.25	90.1	0.98	0.61	8.25

The table reports the mean absolute error (MAE), root mean squared error (RMSE), 1-year accuracy, Pearson correlation coefficient, and bias of Deeplasia’s bone age (BA) estimations relative to the mean of all human raters. The final column (“Mean raters STD”) indicates the mean standard deviation of all raters’ evaluations for each radiograph within the respective subgroup.

**Table 2:** Performance of Deeplasia on different disease subgroups in cohort 2 (LSDs).

Specific subgroup	*N* (images)	Deeplasia MAE	Deeplasia RMSE	Deeplasia 1-year accuracy (%)	Pearson correlation (%)	Bias	STD of experts
Lysosomal disease (unclassified)	43	7.48	9.57	79.0	0.97	2.34	9.39
MPS I	31	7.91	10.59	80.7	0.99	−2.49	10.47
MPS II	19	9.53	13.9	63.0	0.95	−2.51	14.04
MPS III	24	3.87	4.7	100	1.00	2.38	4.14
MPS IV	28	8.25	9.35	78.6	0.99	4.23	8.49
MPS VI	15	7.57	9.9	80.0	0.99	−3.86	9.44
Alpha-mannosidosis	26	5.39	7.47	84.6	0.99	3.86	6.52
Mean of all seven conditions	–	7.14	9.35	80.9	0.98	0.56	10.78
All images in cohort 2	186	7.13	9.56	81.2	0.98	1.04	9.53
Sex: M	117	7.9	10.54	76.1	0.98	1.33	10.51
Sex: W	69	5.81	7.59	89.9	0.99	0.55	7.63
Age: <4 years	46	5.22	6.68	91.3	0.92	1.32	6.62
Age: 4–8 years	48	7.55	9.8	77.1	0.91	2.33	9.62
Age: 8–12 years	26	9.74	13.88	69.2	0.69	−2.76	13.88
Age: >12 years	30	7.56	9.69	76.7	0.98	0	9.85
Age: unknown	36	6.75	8.33	86.1	0.98	2.58	8.03

The table reports the mean absolute error (MAE), root mean squared error (RMSE), 1-year accuracy, Pearson correlation coefficient, and bias of Deeplasia’s bone age (BA) estimations relative to the mean of all human raters. The final column (“Mean raters STD”) indicates the mean standard deviation of all raters’ evaluations for each radiograph within the respective subgroup.

**Table 3:** Comparison of the performance of Deeplasia and individual raters in terms of mean absolute error (MAE), root mean squared error (RMSE), 1-year accuracy, and Pearson correlation coefficient, each calculated relative to the mean of the other four raters in cohort 1 (syndromic and endocrine disorders) for every testing combination.

Testing combination	Reference	Deeplasia				Comparison rater				*p*-value (paired *t*-test)
		MAE	RMSE	1-Year accuracy (%)	Pearson correlation	MAE	RMSE	1-Year accuracy (%)	Pearson correlation	
Deeplasia *vs*. rater 1	Mean of raters 2 and 3	6.25	9.16	87.88	0.98	9.71	13.79	74.65	0.96	<0.0001
Deeplasia *vs*. rater 2	Mean of raters 1 and 3	7.09	9.74	83.74	0.98	8.40	12.09	81.55	0.97	<0.0001
Deeplasia *vs*. rater 3	Mean of raters 1 and 2	6.82	9.10	84.99	0.98	9.44	13.53	76.39	0.96	<0.0001

**Table 4:** Comparison of the performance of Deeplasia and individual raters in terms of mean absolute error (MAE), root mean squared error (RMSE), 1-year accuracy, and Pearson correlation coefficient, each calculated relative to the mean of the other four raters in cohort 2 (LSDs).

Testing combination	Reference	Deeplasia				Comparison rater				*p*-value (paired *t*-test)
		MAE	RMSE	1-Year accuracy (%)	Pearson correlation	MAE	RMSE	1-Year accuracy (%)	Pearson correlation	
Deeplasia *vs*. rater 1	Mean of raters 2, 3, 4, and 5	7.52	10.05	77.96	0.98	8.36	12.17	77.96	0.97	0.0056
Deeplasia *vs*. rater 2	Mean of raters 1, 3, 4, and 5	7.89	10.38	79.03	0.98	10.99	16.03	69.35	0.96	0.0001
Deeplasia *vs*. rater 3	Mean of raters 1, 2, 4, and 5	7.15	9.50	82.26	0.98	8.42	12.56	78.49	0.97	<0.0001
Deeplasia *vs*. rater 4	Mean of raters 1, 2, 3, and 5	7.50	10.10	80.65	0.98	11.45	15.97	66.13	0.95	<0.0001
Deeplasia *vs*. rater 5	Mean of raters 1, 2, 3, and 4	6.96	9.89	82.80	0.98	10.72	15.45	70.97	0.96	<0.0001

**Table 5:** Deeplasia’s predictions and individual as well as mean rater bone age (BA) evaluations for all outlier cases in which the absolute difference between the mean expert BA evaluation and Deeplasia’s prediction exceeded 30 months.

Disorder	Rater 1	Rater 2	Rater 3	Rater 4	Rater 5	Raters mean	Raters STD	SEM	Deeplasia	Difference
Precocious puberty	156	–	39	–	–	97.5	82.7	58.5	155.1	57.6
SHOX deficiency	198	104	192	–	–	164.7	52.6	30.38	206.0	41.3
Ullrich–Turner syndrome	192	138	–	–	–	165	38.2	27.0	132.1	−32.9
Ullrich–Turner syndrome	204	168	–	–	–	186	25.5	18.0	155.6	−30.4
MPS I	90	96	72	66	66	78	14.1	6.29	45.5	−32.5
MPS II	140	138	156	120	108	132.4	18.7	8.36	96.6	−35.8

For the raters’ evaluations, the table additionally reports the standard deviation (STD) and standard error of the mean (SEM). Deeplasia’s estimated BA and the corresponding difference from the expert mean are also shown.

## 3 Results

### 3.1 Cohort 1: syndromic and endocrine disorders

The radiographic images from the first cohort of patients were analyzed by subdividing them into two primary groups based on their clinical presentation: disorders typically associated with accelerated skeletal maturation (see **Figure 1**) and disorders leading to delayed skeletal maturation (see **Figure 2**). In conditions with accelerated growth, Deeplasia demonstrated strong agreement with the expert consensus. Plot showed tight clustering along the line of identity, and Bland–Altman analysis revealed no systematic bias. In disorders with delayed growth, Deeplasia also showed strong agreement, although the mean difference line in the Bland–Altman analysis lay slightly above zero. In total, a minor positive bias of 1.52 months is recorded for all radiographs (see **Table 1**). Outliers, as shown in **Figures 1** and **2**, were defined as cases with a BA difference greater than 30 months between the mean expert evaluation and Deeplasia’s estimation. In total, four outliers were identified: one case of precocious puberty, one of SHOX deficiency, and two cases of Ullrich–Turner syndrome. The absolute BA differences in these cases ranged from 30.4 to 57.6 months. Three of these had been rated by only two raters, the remaining one by all three raters. Moreover, interrater disagreement was observed in these images, with standard deviations of expert evaluations ranging from 25.5 to 82.7 months (almost 7 years). This results in standard error of the means ranging from 18.0 to 58.5 months (see **Table 5**).

**Table 1** summarizes Deeplasia’s performance across diagnostic, sex-, and age-specific subgroups in cohort 1. Across diagnostic categories, MAE values ranged from 5.44 to 9.10 months, RMSE values from 7.21 to 11.11 months, and 1-year accuracies from 70.0% to 92.47%. The lowest MAE values were observed in SHOX deficiency (5.44 months), Noonan syndrome (5.47 months), Silver–Russell syndrome (5.76 months), and congenital adrenal hyperplasia (5.76 months); the highest MAE was seen in pseudohypoparathyroidism (9.10 months). The highest STD of rater BA evaluations was also found for pseudohypoparathyroidism, with 11.88 months.

When stratified by sex, MAE was 6.04 months in female patients and 5.80 months in male patients. Deeplasia’s performance varied across age groups. The model performed best in early childhood (<4 years), with an MAE of 3.76 months, an RMSE of 5.1 months, and a 1-year accuracy of 96.1%. Performance decreased during mid-childhood and early puberty (8–12 years), reaching a maximum MAE of 6.78 months, a corresponding RMSE of 9.02 months, and a minimum 1-year accuracy of 85.8%, before improving again in adolescence (>12 years). Similar trends were observed for the 1-year accuracy and Pearson correlation coefficient. The bias showed an inverse pattern, with the lowest absolute bias of 0.82 months recorded in the 8–12-year age group.

Against individual raters (**Table 3**), Deeplasia achieved lower MAE and RMSE values and higher 1-year accuracies and Pearson correlation coefficients than any single human observer.

### 3.2 Cohort 2: lysosomal storage disorders

**Figure 3** illustrates the agreement between Deeplasia and expert consensus in the lysosomal storage disorder cohort. The scatter plot show a strong linear relationship across the entire BA range, with data points closely aligned along the line of identity and no systematic deviation. The Bland–Altman plot and **Table 2** reveal minimal bias of +1.04 months. Only two outliers were identified in cases of MPS I (BA difference −32.47 months) and MPS II (BA difference −35.80 months). For both cases, Deeplasia’s estimation was outside the range of the five expert evaluations (see **Table 5**).

**Table 2** summarizes Deeplasia’s performance across diagnostic, sex-, and age-specific subgroups in cohort 2. MAE values across diagnostic categories ranged from 3.87 to 9.53 months, RMSE values from 4.70 to 13.90 months, and 1-year accuracies from 63.16% to 100%. The lowest MAE and the highest accuracy were observed in MPS III (MAE 3.87), while MPS II showed the largest deviations (MAE 9.53).

When stratified by sex, MAE was 5.81 months in female patients and 7.90 months in male patients.

Across age groups, the best performance was observed in the youngest children (0–4 years), with an MAE of 5.22 months, an RMSE of 6.68 months, and a 1-year accuracy of 91.3%. After this age range, both MAE and RMSE increased, reaching their peak in children aged 8–12 years, with an MAE of 9.74 months, an RMSE of 13.88 months, and a corresponding minimum 1-year accuracy of 69.2%, before improving again in older age groups.

**Table 4** presents the comparison between Deeplasia and individual human raters in the lysosomal storage disorder cohort. Across all raters, Deeplasia achieved lower MAE and RMSE values and higher 1-year accuracies and Pearson correlation coefficients than any single observer.

## 4 Discussion

Many artificial intelligence methods rely predominantly on the publicly available dataset released in 2017 by the RSNA for their pediatric BA challenge (21). Consequently, published AI methods (28–30, 32) have been trained primarily on images from the general population. Our study, conversely, validates Deeplasia’s performance on these diagnostically complex subgroups.

In a study conducted in 2024, where Deeplasia was first tested on a dysplastic dataset, an MAE of 5.84 months was achieved (34). This generally aligns with the mean MAE over all subgroups of 6.24 months across all conditions in cohort 1 and 7.14 months in cohort 2, respectively. The MAE across all conditions of both cohorts ranged from 3.87 to 9.53 months, with a mean MAE of 6.66 months across all subgroups. The results reflected the diagnostic complexity of these cohorts. Compared to the RSNA dataset [MAE of 3.87 months (34)], Deeplasia achieved a lower level of accuracy on radiographs from children with syndromic and endocrine disorders (cohort 1); predicting bone age was an even greater challenge in patients with lysosomal storage disorders (cohort 2). This indicates that, similar to human raters, assessing bone age using the Greulich and Pyle method is more challenging for Deeplasia on pathological hands than on unaffected hands.

Nevertheless, in both cohorts, Deeplasia consistently achieved higher accuracy across all evaluated metrics (MAE, RMSE, 1-year accuracy, and Pearson correlation coefficients) compared to individual human raters when tested against the mean of other raters (**Tables 3**, **4**)—notably in cohort 2, although a large portion of the radiographs were historical acquisitions (1961–1988), resulting in lower overall image quality that complicated the bone age assessment for all evaluators.

Based on these results, Deeplasia can be considered successfully validated for its generalizability to rare pathological cohorts, which are widely regarded as challenging for current AI-based bone age assessment systems.

Independent of specific radiographs or cohorts, a fundamental difference exists between BA assessment using the human traditional Greulich–Pyle method and Deeplasia. While the first relies on a stepwise, discontinuous process of visual atlas matching, Deeplasia provides a continuous BA prediction down to the monthly and decimal level. This continuous output, which is not bounded by the discrete stages of the GP atlas, constitutes a methodological advantage over the human reference standard. As a result, exact statistical agreement with stepwise human bone age ratings is rarely achievable.

### 4.1 Cohort 1

The subgroup analysis of cohort 1 (**Table 1**) reveals that Deeplasia maintains high and consistent accuracy across most of the syndromic and endocrine disorders, with the MAE tightly clustered in a narrow range of 5.44 to 6.50 months. There was no systematic difference in its performance between conditions characterized by accelerated or delayed skeletal maturation. The only significant deviation was the elevated MAE in pseudohypoparathyroidism (9.10 months), which is most likely attributable to the marked and specific skeletal atypia associated with this rare condition (35). Crucially, even this peak MAE value still represents a very high level of accuracy, confirming that the average prediction error remains well below 12 months. The statistical findings are visually corroborated by the Bland–Altman analysis (**Figure 1B**). This finding suggests that determining bone age based on the Greulich and Pyle atlas is particularly challenging in such cases, as reflected by the fact that this disorder also exhibited the highest standard deviation among rater bone age evaluations within this cohort. Notably, the model achieved excellent results in the remaining dysplastic diseases, despite the known presence of pronounced hand dysplasias in many of these disorders (36, 37).

The most striking observation in the age stratification was a U-shaped accuracy curve. This pattern mirrors clinical experience of human BA evaluation, as these transitional growth phases are methodologically the most challenging to assess.

Furthermore, sex stratification yielded balanced results, confirming the model’s robust performance irrespective of gender. The slightly worse performance for female patients for this cohort is mostly correlated to the large sample size of only female Ullrich–Turner syndrome patients (*n* = 241), which was more difficult to assess (MAE = 6.5 months), while the mean MAE of all eight classes is slightly smaller, with 6.24 months (**Table 1**).

The four outliers in cohort 1 (see **Table 5**), where Deeplasia’s error relative to the mean of the raters exceeded 30 months, are most likely attributable to failures in the human rating process. These could include errors in data entry (e.g., typographical mistakes) or other sources of human error during rating, which led to unusually high standard deviations among rater evaluations and SEMs for these cases. Given the large number of images evaluated in this cohort (*n* = 950), such extreme deviations are expected and do not undermine the overall robustness of the study.

A fast and reliable bone age assessment is highly valuable for the conditions in cohort 1 for several reasons. It can serve as a diagnostic tool, for example, to detect early skeletal maturation and predict adult height in cases of precocious puberty (38), or to identify a typical bone age delay, as seen in Noonan syndrome (37). Another important application is the estimation of remaining growth potential, as well as therapy monitoring during growth hormone treatment in SHOX deficiency (39, 40), Noonan syndrome (41), Silver–Russell syndrome (42), and Turner syndrome (43). In congenital adrenal hyperplasia, treatment with glucocorticoids may accelerate skeletal maturation, which needs to be monitored to optimize growth outcomes (44). Across all these cases, an improved, standardized, and accelerated bone age assessment could significantly enhance patient management and treatment planning.

### 4.2 Cohort 2

Performance across the diverse diagnostic subgroups in cohort 2 (LSDs) reflected the significant impact of phenotypic variability on model accuracy. The higher accuracy observed for MPS III (Sanfilippo syndrome) can be attributed to its relatively mild skeletal involvement and preserved bone proportions, which remain closer to the standard Greulich–Pyle reference morphology (19, 45). In sharp contrast, the lower accuracy for MPS II (Hunter syndrome) is consistent with its pronounced dysostosis multiplex and greater intradiagnostic heterogeneity, which substantially deviates from reference images (33). Similarly, the unclassified LSD group exhibited reduced precision, likely reflecting the broad radiographic and etiologic diversity within that category. Accuracy also showed a decline with age, a pattern associated with the lesser number of available images from older patients and the intrinsic difficulty of evaluating skeletal maturity in later stages of growth when deformities become more pronounced (33). The slight performance differences between sexes may also be explained by the genetic distribution of the included disorders: MPS II is inherited in an X-linked manner and therefore occurs almost exclusively in male patients (46, 47), while all other included lysosomal storage diseases, such as MPS I, III, IV, VI, and alpha mannosidosis (45, 48–52), are autosomal recessive. As a result, the male subgroup contained a higher proportion of severely affected MPS II patients with pronounced skeletal deformities, whereas the female subgroup included a less heterogeneous and overall milder spectrum of diseases.

The outliers in cohort 2 represented instances of disagreement between Deeplasia and all five raters (**Table 5**). However, since Deeplasia overall achieved higher accuracy than any individual rater when compared to the mean of the other raters’ evaluations, this disagreement cannot be considered systematic (**Table 4**). These findings confirm that Deeplasia maintains consistent accuracy and reproducibility even under the challenging skeletal conditions typical of lysosomal storage disorders.

### 4.3 Limitations

A primary constraint is the geographic and ethnic homogeneity of the investigated cohorts, which predominantly reflect the demographic profile of Central Europe (Germany). While the AI model was trained on the globally used RSNA dataset, the generalizability of our specific validation findings to non-European or mixed-ethnicity populations remains limited, suggesting a need for future geographically diverse validation studies (23). Furthermore, the reliance on the Greulich–Pyle method (19) as the reference standard means the AI system inherits the inherent subjectivity and variability of this atlas-based approach, especially in transitional growth phases and in patients with pronounced dysostosis multiplex. Crucially, the absence of a true, independent ground truth means the consensus of human raters, inherently prone to variability, must serve as the reference for the AI system’s performance. Methodological limitations also include the uneven distribution and small sample size in certain rare diagnostic subgroups (e.g., precocious pseudopuberty and MPS VI). These small numbers increase the uncertainty of error metrics in these specific populations and may explain the observed performance drop compared to more prevalent disorders. Additionally, the multicenter nature of the data collection, spanning several decades, introduces unavoidable heterogeneity in image acquisition protocols. In particular, the inclusion of older radiographs in cohort 2 often resulted in image quality and resolution significantly inferior to modern clinical standards, which may attenuate the system’s and the rater’s performance compared to uniformly acquired datasets.

To reduce the impact of these conditions on the total statistics of a cohort, we tried to address this by reporting the mean statistical metrics—MAE, RMSE, 1-year accuracy, and bias—for all conditions in each cohort without disproportionately weighting any condition due to differences in sample size. Finally, as with all artificial intelligence models, there is a lack of explainability in the model’s assessments.

## 5 Conclusion

The present study successfully validated the open-source deep learning system Deeplasia for automated BA assessment on external cohorts of pediatric patients with rare and diagnostically complex growth disorders, including syndromic and endocrine disorders as well as lysosomal storage diseases disorders, and lysosomal storage diseases. From our study, one can conclude that, for Deeplasia, hand radiographs of unaffected children are easier to assess than those of patients with endocrine or syndromic conditions (MAE = 5.95 months across all 950 radiographs), which in turn are easier to evaluate than those of patients with lysosomal storage disorders (MAE = 7.13 months across all 186 images). The same trend was observed for human clinicians, as Deeplasia consistently outperformed individual experts in both cohorts.

While the reliance on the Greulich–Pyle method as the reference standard and the geographic homogeneity of the cohorts remain limitations, Deeplasia offers a promising path toward standardizing BA assessment in pediatric practice.

## Data Availability

The raw data supporting the conclusions of this article will be made available by the authors, without undue reservation.
